# Do the unusual resignations of independent directors affect auditors’ professional judgment?

**DOI:** 10.1371/journal.pone.0304585

**Published:** 2024-06-20

**Authors:** Li Zhang, Zigui Li

**Affiliations:** TianJin University of Commerce, TianJin, China; Wroclaw University of Environmental and Life Sciences: Uniwersytet Przyrodniczy we Wroclawiu, POLAND

## Abstract

In Kangmei’s first trial judgment, where the independent directors faced significant joint and several liabilities, it triggered a "wave" of resignations among independent directors of listed companies. Auditors auditing financial reports of these companies might consider this a significant signal, raising the question: does this signal influence their professional judgment? The study examines the relationship between the resignation of independent directors and auditors’ professional judgment in A-share listed companies, following Kangmei’s initial trial. This examination is conducted across three dimensions: audit pricing, audit input, and audit opinion.The findings indicate that the unusual resignations of independent directors prompt uditors to pay increased attention to the risk of material misstatements by clients, primarily reflected in audit pricing. However, these resignations do not significantly impact audit input or the judgment of audit opinions. Furthermore, this research enriches the existing literature on audit pricing and the role of independent directors, while also unveiling the specific pathways through which the departure of independent directors impacts auditors’ professional judgment.

## Introduction

The role and behavior of independent directors in corporate governance are of paramount importance, serving as a supervisory mechanism to ensure the fairness and transparency of the decision-making process within management teams. However, in recent years, the phenomenon of resignations among independent directors has drawn widespread public attention and academic discussion, especially when such resignations occur following legal disputes or corporate crises. This behavior is viewed as a potential key indicator affecting corporate governance and market confidence [1–4]. Consequently, the primary question of this study is to explore whether the abnormal resignation of independent directors influences the professional judgment of auditors and to attempt to decipher the specific pathways and mechanisms of this influence.

This study delves into the impact of abnormal resignations of independent directors on the professional judgment of auditors, aiming to investigate whether such resignations alter auditors’ attitudes towards audit pricing, audit inputs, and audit opinions. By integrating theoretical analysis and empirical testing, this research seeks to provide deeper insights into the roles and effects of independent directors while offering an empirical foundation for audit practices. The theoretical basis of this study encompasses literature on corporate governance, audit independence, and risk management. Resignations of independent directors, as internal monitors, may be interpreted by the market as signals of internal control weaknesses or potential corporate governance issues, subsequently affecting external auditors’ assessments of the firm’s financial condition [5, 6]. Moreover, from the perspective of the audit market, the abnormal resignation of independent directors may increase the professional risks faced by auditors, thereby influencing their work methods and decision-making [7, 8]. Through an in-depth analysis of these dynamics, this research aims to reveal the intrinsic link between the abnormal resignation behavior of independent directors and the professional judgment of auditors.

To ensure the contribution and novelty of the research findings, this study will conduct a critical review and analysis of existing literature, especially those exploring the relationship between the resignation of independent directors and audit quality. Although previous studies have explored the market reaction and economic consequences of independent director resignations, research on how these resignations specifically affect the audit process remains relatively limited. This study aims to fill this gap by examining the relationship between independent director resignations and the professional judgment of auditors in the Chinese A-share market, providing new perspectives on how auditors respond to changes in corporate governance. Methodologically, this paper will adopt a quantitative analysis approach, systematically analyzing the relationship between independent director resignations and audit pricing, audit inputs, and audit opinions based on data from Chinese A-share listed companies. By constructing a multiple regression model and controlling for relevant variables, this research aims to objectively assess the actual impact of independent director resignations on the professional judgment of auditors. Additionally, case study methods will be employed to deeply explore how the resignation of independent directors in specific events affects auditors’ behaviors and decision-making.

By analyzing the case of Kangmei Pharmaceutical’s first-instance judgment and the subsequent resignation phenomenon of its independent directors, this study will provide new insights into the connection between the resignation behavior of independent directors and the professional judgment of auditors. Through empirical research, this paper aims to reveal how the abnormal resignation of independent directors affects audit quality and efficiency by influencing auditors’ risk perceptions and decision-making processes. Moreover, the findings of this study will provide empirical support for corporate governance, audit practices, and regulatory policy formulation, especially in strengthening the role of independent directors and improving audit quality.

The structure of this paper is as follows: It begins with a literature review analyzing the relationship between the resignation of independent directors and auditors’ risk perception, and proposes research hypotheses. The next part details the research design, including sample selection, data sources, definitions of variables, and model settings. This is followed by a presentation of the research findings through descriptive statistics, correlation analysis, multiple regression analysis, and an interpretation and analysis of the results. Finally, the paper discusses the significance of the study, provides a summary, highlights the limitations of this research, and outlines directions for future work.

Path analysis and formulation of research hypotheses: the impact of independent directors’ abnormal resignations on auditors’ professional judgment

### (i) The influence of independent directors’ abnormal resignations on auditors’ risk perception

From a regulatory perspective, both independent directors and auditors serve as supervisors of listed companies, with overlapping areas of oversight [9, 10]. Independent directors have a regulatory duty over the financial information of listed companies, which in practice manifests as supervision over the company’s operating condition, significant related party relationships and transactions, loans or other financial transactions between shareholders, actual controllers and their affiliated enterprises and the listed company, external guarantees, changes in accounting policies, accounting estimates, or major accounting error corrections not due to changes in accounting standards, internal controls, and significant asset restructurings, among others [11, 12]. Auditors, when auditing the financial statements of listed companies, focus their examination and professional judgment on the financial data resulting from these issues [13–15]. Therefore, following the first-instance judgment in the Kangmei case, the resignation of independent directors could likely be due to their failure to effectively exercise their supervisory functions during their tenure. When auditors receive the signal of an independent director’s resignation, they may pay closer attention to matters related to the duties of independent directors that affect the financial statements in the audit process.

**Fig 1:**
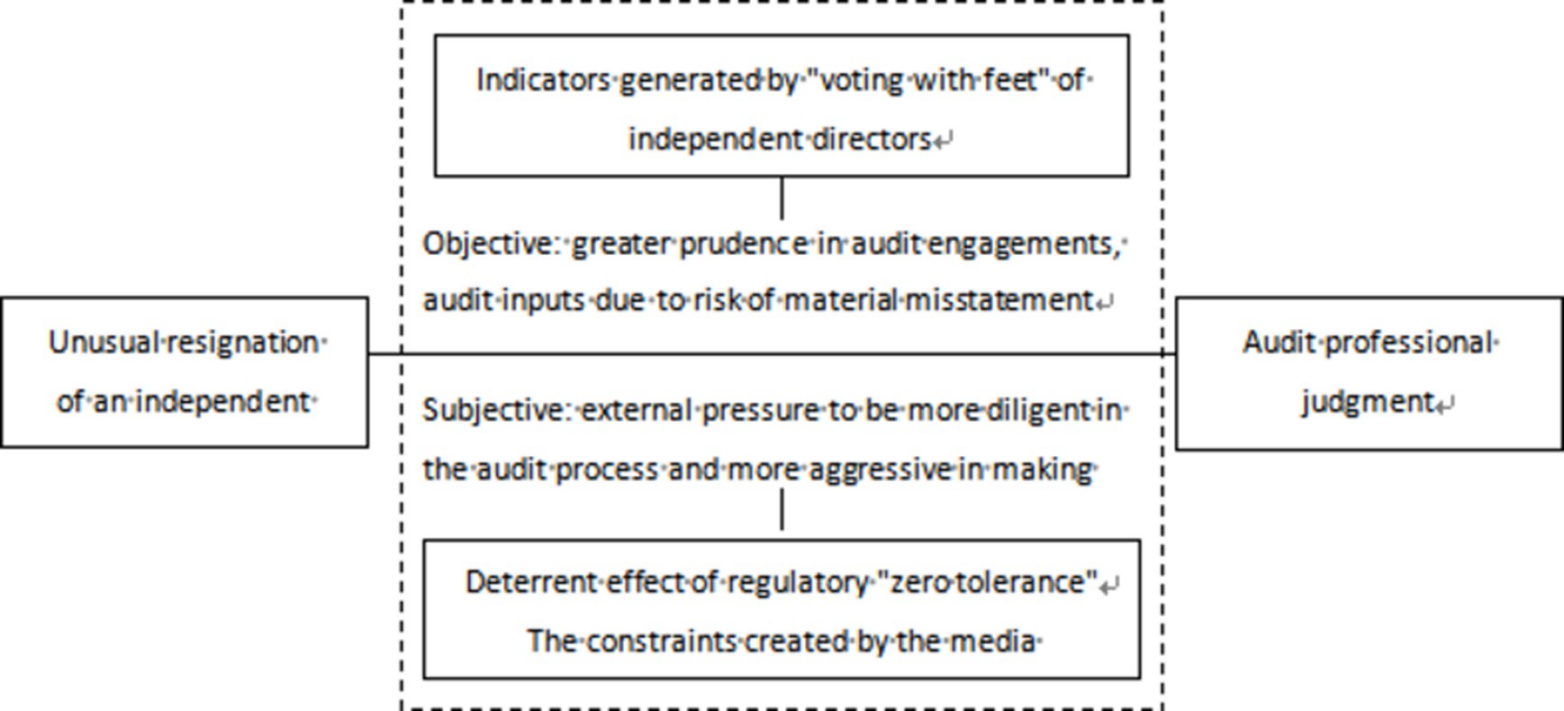
Path of the impact of abnormal resignation of independent directors on audit professional judgment.

Independent directors, as regulators within corporate governance, are the providers of information, while certified public accountants, as external supervisors, are the recipients of this information. The approval or disapproval votes cast by independent directors on certain issues are significant pieces of information for auditors during the corporate governance process [16]. However, from the perspective of the appointment of independent directors in China, their independence is somewhat constrained to a certain extent, making it difficult for them to be effective. In most cases, they choose to "vote with their feet" [17–19]. Starting from the reputation mechanism, if the financial condition of the company is good and the risk level is low without any other special risk factors, independent directors generally will not choose to resign actively. However, when independent directors perceive potential risks to the company and their independence is restricted, the risks of continuing in their role far outweigh the benefits [20], leading independent directors to more frequently choose resignation as a risk avoidance strategy [21, 22]. Similarly, when certified public accountants learn of the resignation of independent directors following the Kangmei first-instance judgment, they may pay more attention to economic matters related to the duties of independent directors during the audit process to obtain more sufficient and appropriate audit evidence, thereby providing a basis for issuing the final audit opinion. It can be inferred that, objectively, the resignation of independent directors can impact auditors’ professional judgment in business acceptance and audit input processes (as illustrated in Fig 1).

### (ii) Enhanced ’zero tolerance’ government regulations and increased audit diligence

With the implementation of the new Securities Law, the perception of pressure on external audits has significantly increased, especially in the aftermath of the Kangmei Pharmaceuticals case. When a listed company’s independent directors resign without cause, such events may be perceived as indicators of a weakened corporate governance structure and reduced transparency of information, according to Agency Theory [23] and Information Asymmetry Theory [24]. This perception, in turn, increases the risk of significant misstatements in the company’s financial reporting. As key figures in corporate governance, the resignation of independent directors may imply an intensification of agency problems between management and shareholders, as well as a worsening of the information asymmetry between shareholders and the external market. Consequently, these events prompt auditors to take additional measures to reduce audit risk, including increasing the depth and breadth of audit procedures to ensure the accuracy of financial reports [25].

According to previous research, the resignation of independent directors could be interpreted as a precursor to internal control deficiencies, thereby increasing the likelihood of financial misstatements [26]. In response to this potential risk, auditors enhance audit quality by increasing audit inputs and executing more audit procedures to detect significant misstatements and omissions in the financial statements as thoroughly as possible [27]. Furthermore, considering the Risk Premium Theory, auditors facing companies with a high risk of misstatement may demand higher audit fees as compensation for the risk [28]. Additionally, to strengthen their defense in potential future legal litigations and mitigate potential legal liabilities, auditors may be inclined to issue more non-standard audit opinions.

In summary, based on the implementation of the new Securities Law and the cautionary tale of the Kangmei Pharmaceuticals incident, auditors should adopt a more cautious audit strategy for listed companies experiencing unexplained resignations of independent directors. By increasing audit inputs, raising audit fees, and adopting more conservative audit opinions when necessary, auditors aim to detect and report potential financial errors to the fullest extent, thereby avoiding future legal risks and professional liabilities.

### (iii) Increased media attention and pressure for audit opinion judgments

In contemporary society, media serves as a critical bridge between corporations and public investors, playing a significant role in supervising companies by disseminating information [29]. According to related research, companies that receive negative media coverage are more likely to become the focus of key stakeholders such as regulatory bodies and investors. This phenomenon not only increases the audit risk faced by auditors but also raises the potential for audit litigation [30, 31]. It is evident that the nature and scope of media coverage profoundly impact auditors’ professional judgments and the choice of audit strategies. In response to risks induced by media coverage, auditors may adopt a series of measures, including but not limited to, adjusting audit processes, expanding the scope of the audit, and increasing the time and resources devoted to audit activities. These measures aim to mitigate the increased risks associated with negative coverage, consequently leading to a significant rise in audit fees. Additionally, when auditors confront media reports, they actively seek and review relevant information. This behavior may alter their perceptions of "information possession" and "risk weight judgment," potentially exacerbating cognitive biases about listed companies and increasing the likelihood of issuing non-standard audit opinions [32]. Specifically, the wave of resignations by independent directors following the Kangmei Pharmaceuticals judgment and the subsequent extensive media attention significantly heightened public awareness of such events. In this context, some risk-averse accounting firms might adopt a cautious stance towards accepting companies that have experienced resignations of independent directors. The increased caution exercised by auditors during the audit process and the heightened aggressiveness in issuing audit opinions can be seen as strategies to avoid public condemnation.

In summary, auditors auditing companies with independent director resignations post-Kangmei’s first trial may adjust their professional judgments in audit pricing, audit inputs, and audit opinion decision-making to mitigate potential future risks. Accordingly, this study proposes the following hypotheses:

H1: Ceteris paribus, auditors are likely to increase the audit pricing for companies experiencing unusual resignations of independent directors.
H2: Ceteris paribus, auditors will increase their inputs in companies with unusual independent director resignations, potentially leading to audit delays.
H3: Ceteris paribus, auditors are more inclined to issue non-standard audit opinions for companies with unusual independent director resignations.

## Research design

### (i) Sample selection and data sources

This study selects A-share listed companies from January to December 2021 as the initial sample, focusing on whether there is an abnormal resignation of independent directors within the year as the subject of research. Data on abnormal resignations of independent directors were manually collated through the Juchao Information Website, while other related data were obtained from the China Stock Market & Accounting Research (CSMAR) database. The initial sample excluded companies in the financial and insurance sectors, ST and *ST companies, companies that have been delisted, and samples missing control variables and related data. After these exclusions, a total of 3,469 annual company samples were obtained. Continuous variables were subjected to winsorization at the 99% and 1% levels to mitigate the influence of outliers.

### (ii) Definition of variables and modeling

In order to investigate the effects of abnormal departure of independent directors on audit fees, audit inputs, and types of audit opinions, this study builds the following models, respectively:

$$\begin{array}{l} {\text{AFEE}}_{\text{i,t}}=\alpha_{0}+\alpha_{1}{\text{Resign}}_{\text{i,t}}+\alpha_{2}{\text{SIZE}}_{\text{i,T}}+\alpha_{3}{\text{LEV}}_{\text{i,t}}+\alpha_{4}{\text{ROA}}_{\text{i,t}}+\alpha_{5}{\text{LOSS}}_{\text{i,t}}+\alpha_{6}{\text{CUR}}_{\text{i,t}}+\alpha_{7}{\text{INV}}_{\text{i,t}}+\alpha_{8}{\text{REC}}_{\text{i,t}}+\alpha_{9}{\text{GROWTH}}_{\text{i,t}}\\ +\alpha_{10}{\text{SOE}}_{\text{i,t}}+\alpha_{11}\text{BIG}4_{\text{i,t}}+\alpha_{12}{\text{LOPINION}}_{\text{i,t}}+\sum \text{YEAR}+\sum \text{IND}+\varepsilon \end{array}$$ (1)

$$\begin{array}{l} {\text{AINVEST}}_{\text{i,t}}=\beta_{0}+\beta_{1}{\text{Resign}}_{\text{i,t}}+\beta_{2}{\text{SIZE}}_{\text{i,T}}+\beta_{3}{\text{LEV}}_{\text{i,t}}+\beta_{4}{\text{ROA}}_{\text{i,t}}+\beta_{5}{\text{LOSS}}_{\text{i,t}}+\beta_{6}{\text{CUR}}_{\text{i,t}}+\beta_{7}{\text{INV}}_{\text{i,t}}+\beta_{8}{\text{REC}}_{\text{i,t}}+\beta_{9}{\text{GROWTH}}_{\text{i,t}}\\ +\beta_{10}{\text{SOE}}_{\text{i,t}}+\beta_{11}\text{BIG}4_{\text{i,t}}+\beta_{12}{\text{LOPINION}}_{\text{i,t}}+\sum \text{YEAR}+\sum \text{IND}+\varepsilon \end{array}$$ (2)

$$\begin{array}{l} {\text{AOP}}_{\text{i,t}}=\gamma_{0}+\gamma_{1}{\text{Resign}}_{\text{i,t}}+\gamma_{2}{\text{SIZE}}_{\text{i,T}}+\gamma_{3}{\text{LEV}}_{\text{i,t}}+\gamma_{4}{\text{ROA}}_{\text{i,t}}+\gamma_{5}{\text{LOSS}}_{\text{i,t}}+\gamma_{6}{\text{CUR}}_{\text{i,t}}+\gamma_{7}{\text{INV}}_{\text{i,t}}+\gamma_{8}{\text{REC}}_{\text{i,t}}+\gamma_{9}{\text{GROWTH}}_{\text{i,t}}\\ +\gamma_{10}{\text{SOE}}_{\text{i,t}}+\gamma_{11}\text{BIG}4_{\text{i,t}}+\gamma_{12}{\text{LOPINION}}_{\text{i,t}}+\sum \text{YEAR}+\sum \text{IND}+\varepsilon \end{array}$$ (3)

Models (1), (2), and (3) are used to test the effects of abnormal departure of independent directors on audit fees, audit inputs, and types of audit opinions, respectively, with OLS regression for models (1) and (2) and Logit regression for model (3).

Here, the subscript ’i’ denotes the individual firm, and ’t’ denotes the year. The dependent variable ’resign’ represents abnormal departures of independent directors, with the dummy variable ’resign’ assigned a value of 1 for departures and 0 otherwise. The explanatory variables for the three models include audit fees (’AFEE’), audit inputs (’INVEST’), and the type of audit opinion issued by the auditor (’OPINION’). ’AFEE’ is defined as the natural logarithm of the total audit fees for the year. ’INVEST’ is determined by the natural logarithm of the number of calendar days between the balance sheet date and the date of signing the audit report. For ’OPINION’, the variable is assigned a value of 0 if the auditor issued a standard unqualified audit opinion for the year, and 1 otherwise.

To minimize the impact of omitted variables on the empirical results, this study includes firm size (SIZE), gearing ratio (LEV), corporate profitability (ROA), loss in the current year (LOSS), current ratio (CUR), inventory ratio (INV), accounts receivable ratio (REC), firm growth (GROWTH), and the nature of ownership (SOE) as control variables. Additionally, changes in accounting firms (ACH), being audited by the ’Big 4’ (BIG4), and changes in audit opinion (LOPINION) are included as control variables. To control for year and industry effects, this study also incorporates year dummy variables (YEAR) and industry dummy variables (INDUS). Specific definitions for each variable are provided in Table 1.

**Table 1:** Definition of variables.

Variable type	Name and Code	define
explanatory variable	Audit fees (AFEE)	Natural logarithm of total audit costs
	Audit delays (AINVEST)	Ln (number of calendar days between balance sheet date and audit report date)
	Audit opinion (AOP)	Dummy variable that takes the value of 0 if the auditor issued a standard unqualified audit opinion for the year, and 1 otherwise
explanatory variable	Resignation of Independent Director (Resign)	Dummy variable that takes the value of 1 if there is an abnormal departure of an independent director in a listed company and 0 otherwise
control variable	Company size (Size)	Natural logarithm of total assets at the end of the period
	Level of indebtedness (LEV)	Gearing ratio, total liabilities at end of period divided by total assets at end of period
	Profitability (ROA)	Return on assets, net profit divided by total assets at the end of the period
	Whether a loss has been incurred (LOSS)	Dummy variable that takes the value of 1 if the net profit for the year is negative and 0 otherwise
	Current ratio (CUR)	Current assets at end of period divided by total assets at end of period
	Inventory as a percentage (INV)	Closing inventory divided by closing total assets
	Accounts receivable as a percentage (REC)	Accounts receivable at end of period divided by total assets at end of period
	Business Growth (GROWTH)	Operating income growth rate, the growth rate of the company’s current year’s operating income relative to the previous year’s operating income
	Change of Accounting Firm (ACH)	If the company has changed its accounting firm, the value is 1, otherwise it is 0.
	Nature of ownership of enterprise (SOE)	1 for state-owned enterprises, 0 otherwise
	Whether audited by Big 4 (BIG4)	Audit by the "Big Four" takes the value of 1, otherwise 0
	Prior period audit opinion (LOPINION)	A standard unqualified audit opinion has been issued for the previous period, which takes the value of 0, otherwise it takes the value of 1.
	Year (YEAR)	Year dummy variable to control for the effect of the year factor
	Industry (IND)	Industry dummy variables to control for the effect of industry factors

## Empirical results and analysis

### (i) Descriptive statistics

Based on the descriptive statistical results in Table 2, we can delve further into the characteristics of each variable as they manifest in the sample. Firstly, the proportion of abnormal resignations among independent directors is 13.0%, which may indicate potential issues in oversight and independence, meriting further exploration of its impact on corporate governance and risk management. The average audit fee (AFEE) is 13.86, equivalent to approximately 1.0455 million yuan, demonstrating significant pricing diversity in the audit market. The 25th and 75th percentiles are 13.430 and 14.221, corresponding to about 680,100 yuan and 1.5 million yuan, respectively. The maximum and minimum values are 11.775 and 17.548, equivalent to about 130,000 yuan and 43.3146 million yuan, respectively, showing a variance of 43.1846 million yuan. This indicates substantial differences in audit fees charged by firms to different listed companies, reflecting potentially varying audit service quality, company size, business complexity, or risk levels. The distribution of audit fees also reveals the demand and supply conditions for audit services in the market, as well as differences in pricing strategies among audit firms.

**Table 2:** Descriptive statistics.

VarName	Mean	SD	Median	P25	P75	Min	Max.
Resign	0.13	0.34	0.000	0.000	0.000	0.000	1.000
AFEE	13.86	0.64	13.764	13.430	14.221	11.775	17.548
AINVEST	4.62	0.17	4.700	4.489	4.754	2.773	5.112
AOP	0.02	0.15	0.000	0.000	0.000	0.000	1.000
SIZE	19.74	6.64	21.826	20.939	22.843	2.925	28.543
LEV	0.42	0.28	0.408	0.259	0.553	0.019	9.429
ROA	0.04	0.11	0.043	0.018	0.074	-3.698	0.604
LOSS	0.12	0.32	0.000	0.000	0.000	0.000	1.000
CUR	2.62	2.87	1.769	1.242	2.956	0.015	78.512
INV	0.13	0.11	0.110	0.057	0.174	0.000	0.818
REC	0.12	0.10	0.106	0.047	0.178	0.000	0.640
GROWTH	-0.28	8.33	0.108	-0.270	0.425	-359.777	109.528
SOE	0.29	0.45	0.000	0.000	1.000	0.000	1.000
ACH	0.08	0.27	0.000	0.000	0.000	0.000	1.000
BIG4	0.07	0.26	0.000	0.000	0.000	0.000	1.000
LOPINION	0.03	0.16	0.000	0.000	0.000	0.000	1.000

The mean value for audit input (AINVEST) is 4.62, with an average audit delay of 102 days, which could affect the timeliness of financial information and audit quality. The 25th and 75th percentiles are 4.489 and 4.754, equivalent to approximately 89 and 116 days, respectively. The maximum and minimum values are 5.112 and 2.773, equivalent to 166 days and 16 days, respectively, showing a significant difference of 150 days. This indicates clear differences in audit input for different companies, where a wide range of fluctuations may suggest significant variances in operational and financial reporting complexities requiring different levels of time and resources from auditors.

The average audit opinion (AOP) value is 0.02, meaning that 2% of companies received a non-standard audit opinion. Although this percentage is low, it remains significant because non-standard audit opinions are usually associated with financial health, internal control, or compliance issues, significantly impacting investor confidence and market participants.

Other variables, such as company size (SIZE), financial leverage (LEV), return on assets (ROA), proportion of losses (LOSS), current ratio (CUR), inventory ratio (INV), receivables ratio (REC), growth (GROWTH), proportion of state-owned enterprises (SOE), accounting conservatism (ACH), proportion of audits by Big Four firms (BIG4), and audit opinion delay (LOPINION), reflect the diverse financial and operational characteristics of the sample companies. The distribution and statistical characteristics of these variables lay the foundation for a deeper exploration of the financial conditions, market performance, and risk-taking of the firms. These descriptive statistics offer a wealth of information for further analysis, helping to gain a deeper understanding of the operational conditions and market environment of the sample companies.

### (ii) Correlation coefficient analysis

According to the Spearman correlation coefficient results in Table 3, the correlation coefficient between audit fees (AFEE) and the resignation of independent directors (Resign) is 0.04, which is significant at the 5% level. This suggests that the resignation of independent directors leads to an increase in audit fees to some extent. The correlation coefficient between audit delay (AINVEST) and the resignation of independent directors (Resign) is 0.05, significant at the 1% level, indicating that the resignation of independent directors somewhat causes auditors to increase audit efforts, leading to audit delays. The correlation coefficient between the type of audit opinion (AOP) and the resignation of independent directors (Resign) is 0.15, significant at the 1% level, suggesting that the resignation of independent directors can lead auditors to issue non-standard audit opinions.

**Table 3:** Correlation coefficient matrix-Spielman.

	AOP	AFEE	AINVEST	Resign	SIZE	LEV	ROA	LOSS	CUR	INV	REC	GROWTH	SOE	ACH	BIG4	LOPINION
AOP	1															
AFEE	0.03**	1														
AINVEST	0.15***	0.04**	1													
Resign	0.15***	0.04**	0.05***	1												
SIZE	-0.09***	0.50***	-0.08***	-0.59***	1											
LEV	0.09***	0.40***	0.04***	0.06***	0.36***	1										
ROA	-0.17***	-0.10***	-0.20***	-0.11***	0	-0.42***	1									
LOSS	0.25***	0.06***	0.20***	0.11***	-0.07***	0.20***	-0.55***	1								
CUR	-0.09***	-0.39***	-0.04**	-0.06***	-0.35***	-0.85***	0.37***	-0.20***	1							
INV	-0.02	0	0	0.01	-0.01	0.17***	-0.03*	-0.03*	0	1						
REC	-0.02	-0.09***	0.03*	0.01	-0.19***	0.04***	-0.07***	0.02	0.09***	0.09***	1					
GROWTH	-0.09***	0.04**	-0.20***	0.09***	0.02	-0.04**	0.51***	-0.44***	0.01	0.01	-0.10***	1				
SOE	-0.04***	0.10***	-0.14***	0.01	0.29***	0.25***	-0.10***	-0.03	-0.25***	-0.03**	-0.25***	0.09***	1			
ACH	0.11***	0	0.03*	0.06***	-0.02	0.04**	-0.08***	0.06***	-0.04**	-0.02	-0.02	-0.01	0.08***	1		
BIG4	-0.02	0.30***	-0.11***	0.07***	0.14***	0.10***	-0.01	-0.01	-0.10***	-0.04**	-0.09***	0.02	0.12***	0.05***	1	
LOPINION	0.54***	0.03**	0.12***	0.16***	-0.12***	0.08***	-0.14***	0.20***	-0.09***	-0.04**	-0.01	-0.07***	-0.03	0.11***	-0.04**	1

*** p<0.01

** p<0.05

* p<0.1

In summary, the higher the resignation rate of independent directors, the more audit resources auditors invest, leading to higher audit fees, potential audit delays, and a tendency for auditors to issue non-standard audit opinions. This aligns with the expectations of this study’s hypothesis. However, to ascertain whether there is a strict causal relationship between audit fees, audit delays, audit opinions, and the resignation of independent directors, further multivariate regression analysis is needed, controlling for other related variables.

### (iii) Multiple regression analysis

The OLS regression results presented in column (1) of Table 4 for audit fees and the resignation of independent directors show a positive correlation between audit fees and the resignation of independent directors, with a coefficient of 6.486, significant at the 1% level. This outcome is consistent with Hypothesis H1, indicating that, all else being equal, companies experiencing abnormal resignations of independent directors incur higher audit fees.

**Table 4:** Multiple regression results.

	(1)	(2)	(3)
	AFEE	AINVEST	AOP
Resign	6.486***	0.066	0.562
	(47.03)	(1.22)	(0.20)
SIZE	0.338***	0.003	0.013
	(47.94)	(1.15)	(0.09)
LEV	0.092**	-0.030*	0.609
	(2.21)	(-1.82)	(0.74)
ROA	0.107	-0.094**	-4.002**
	(1.05)	(-2.37)	(-2.38)
LOSS	0.083***	0.057***	1.435***
	(3.07)	(5.37)	(3.68)
CUR	-0.011***	-0.000	-0.072
	(-3.56)	(-0.14)	(-0.62)
INV	-0.174***	0.042*	2.377**
	(-2.67)	(1.65)	(2.46)
REC	0.186**	0.020	-0.169
	(2.44)	(0.68)	(-0.12)
GROWTH	0.001	-0.000	0.036*
	(0.81)	(-0.86)	(1.89)
SOE	-0.194***	-0.040***	-0.871**
	(-11.00)	(-5.85)	(-2.20)
ACH	-0.069**	0.012	0.740*
	(-2.51)	(1.10)	(1.81)
BIG4	0.554***	-0.067***	-0.164
	(18.73)	(-5.83)	(-0.22)
LOPINION	0.099**	0.045**	3.898***
	(2.01)	(2.36)	(11.25)
_cons	6.265***	4.561***	-6.160*
	(40.52)	(75.76)	(-1.95)
N	3469	3469	3469
YEAR	Yes	Yes	Yes
IND	Yes	Yes	Yes
R^2^	0.560	0.050	
ar2			

*** p<0.01

** p<0.05

* p<0.1

Column (2) displays the OLS regression results for audit delays and the resignation of independent directors, revealing that the relationship between audit delay and the resignation of independent directors is not significant. This finding does not validate Hypothesis H2, suggesting that while the abnormal resignation of independent directors may impact the auditors’ input, it does not lead to audit delays. Instead, auditors issue audit opinions within the normal timeframe to avoid attracting undue attention.

Column (3) presents the Logit regression results for audit opinion types and the resignation of independent directors, showing that the relationship between audit opinions and the resignation of independent directors is not significant. This outcome does not support Hypothesis H3, implying that although auditors might consider the resignation of independent directors when issuing audit opinions, the final type of audit opinion primarily depends on the financial condition of the audited entity rather than the resignation status of independent directors.

These results collectively provide insights into how auditors respond to the resignation of independent directors. While such resignations lead to increased audit fees, likely due to perceived higher risks or additional work required to assess the implications of these resignations on corporate governance, they do not necessarily result in audit delays or a greater likelihood of issuing non-standard audit opinions. This indicates that auditors might adjust their work in response to perceived risks without compromising the timeliness of their audit processes or the basis on which they issue opinions, focusing instead on the audited entity’s financial health.

A further comparison of the financial indicators of companies that experienced unusual resignations of independent directors in 2021, as presented in Table 5, reveals that for companies with both resignations of independent directors and non-standard audit opinions, the average values of financial indicators—namely current asset turnover, total asset turnover, current ratio, cash flow ratio, return on net assets, and net operating margin—are all lower than those for companies with standard audit opinions. Additionally, the relatively high gearing ratio and negative figures for cash flow ratio, return on net assets, and net operating margin contribute to the frequent inclusion of the "going concern" qualification in the basis for their non-standard audit opinions. This indicates that while the unusual resignation of independent directors may signal certain concerns to the CPA, the ultimate determination of the audit opinion is still derived from the CPA’s holistic assessment of the risk of material misstatement in the audited entity’s financial statements.

**Table 5:** Analysis of financial indicators of companies resigned by sole managers.

Type of audit opinion	managerial ability		solvency			profitability	
	Current asset turnover ratio	Total asset turnover	current ratio	cash flow ratio	gearing	return on net assets	do business net interest rate
Non-standard audit opinion	0.88	0.36	1.22	-0.03	0.97	-1.10	-4.06
Standard unqualified audit opinion	1.19	0.65	2.52	0.20	0.43	0.04	0.05

### (iv) Robustness tests

#### 1. Endogeneity test

This study investigates the causal link between the abnormal resignations of independent directors and audit decisions, acknowledging that this relationship may be obscured by endogeneity concerns[33, 34]. For instance, firms experiencing abnormal resignations of independent directors and concurrently receiving non-standard audit opinions typically exhibit weaker financial performance. Such firms may be more disposed to engage auditors perceived as less prestigious, who might be more likely to curtail audit efforts, decrease audit fees, and issue standardized unqualified opinions, thereby introducing a self-selection bias.

Consequently, this study employs propensity score matching (PSM) to address potential endogeneity issues. To ensure comparability between the treatment and control groups and to more effectively address the endogeneity concern, this study selects a set of covariates for matching: gearing ratio (LEV), corporate profitability (ROA), current year losses (LOSS), current ratio (CUR), enterprise growth (GROWTH), accounting firm change (ACH), auditing by the "Big 4" (BIG4), and the prior period’s audit opinion (LOPINION). These covariates were utilized to generate matched samples via the radius matching method (r = 0.05). The resultant matched samples from both the treatment and control groups showed no significant differences, meeting the balance test criteria.

Regression analyses were performed on the PSM-matched samples, with the findings presented in Table 6. These analyses confirm that the results using the propensity score matched samples align with those from the initial regression (Table 4), indicating that the resignation of an independent director significantly increases audit fees, yet has no significant impact on audit delays or the issuance of non-standard audit opinions. This further indicates that the abnormal resignation of independent directors conveys certain signals to auditors, prompting them to increase audit fees as compensation for potential future risks associated with accepting an audit engagement. Moreover, auditors are less inclined to encounter audit delays, which could draw unwarranted regulatory scrutiny. Likewise, the likelihood of issuing a non-standard audit opinion does not increase due to the atypical resignations of independent directors from a listed company.

**Table 6:** PSM sample regression results.

	(1)	(2)	(3)
	AFEE	AINVEST	AOP
Resign	6.319***	0.035	0.515
	(44.26)	(0.64)	(0.18)
SIZE	0.330***	0.002	0.012
	(45.11)	(0.56)	(0.08)
LEV	0.246***	-0.002	0.598
	(4.48)	(-0.08)	(0.71)
ROA	0.080	-0.255***	-3.769**
	(0.53)	(-4.32)	(-2.15)
LOSS	0.065**	0.027**	1.419***
	(2.10)	(2.25)	(3.62)
CUR	-0.006*	0.001	-0.069
	(-1.78)	(0.76)	(-0.59)
INV	-0.229***	0.029	2.440**
	(-3.44)	(1.12)	(2.53)
REC	0.120	0.001	-0.066
	(1.55)	(0.03)	(-0.05)
GROWTH	0.001	-0.002**	0.030
	(0.35)	(-2.29)	(1.28)
SOE	-0.199***	-0.040***	-0.858**
	(-11.27)	(-5.84)	(-2.18)
ACH	-0.064**	0.011	0.739*
	(-2.28)	(1.02)	(1.80)
BIG4	0.555***	-0.067***	-0.168
	(18.82)	(-5.78)	(-0.22)
LOPINION	0.103**	0.040**	3.843***
	(2.03)	(2.04)	(10.97)
_cons	6.404***	4.598***	-6.145*
	(40.57)	(74.67)	(-1.95)
N	3425	3425	3425
IND	Yes	Yes	Yes
YEAR	Yes	Yes	Yes
R^2^	0.563	0.056	
ar2			

*** p<0.01

** p<0.05

* p<0.1

#### 2. Substitution of explanatory variables

The OLS regression was rerun, substituting the audit fee metric from Ln (current audit fee) to Ln (current audit fee/total assets). Additionally, the audit delay variable was recalibrated from Ln (the number of calendar days from the balance sheet date to the audit report date) to the raw count of days, without applying the logarithmic transformation. Furthermore, the audit opinion was redefined in the OLS regression from the initial dummy variable to an ordinal variable, whereby a standard unqualified opinion is assigned a value of 0, an unqualified opinion with an emphasis of matter paragraph is assigned a value of 1, a qualified opinion, with or without an additional matter or emphasis paragraph, is assigned a value of 2, and an opinion indicating an inability to express a view is assigned a value of 3. The findings from the ordered Logit regression analysis are presented in Table 7.

**Table 7:** Regression results with replacement of explanatory variables.

	(1)	(2)	(3)
	AFEE_New	AINVEST_New	AOP_New
Resign	6.514***	3.372	0.334
	(47.25)	(0.70)	(0.13)
SIZE	-0.660***	0.149	-0.001
	(-93.53)	(0.60)	(-0.00)
LEV	0.094**	-2.417*	1.231**
	(2.25)	(-1.66)	(2.08)
ROA	0.088	-7.927**	-1.427*
	(0.86)	(-2.23)	(-1.69)
LOSS	0.084***	5.959***	1.759***
	(3.09)	(6.31)	(5.34)
CUR	-0.010***	-0.033	-0.078
	(-3.40)	(-0.32)	(-0.73)
INV	-0.179***	3.863*	1.702*
	(-2.75)	(1.70)	(1.79)
REC	0.201***	1.403	-1.507
	(2.64)	(0.53)	(-1.06)
GROWTH	0.001	-0.028	0.027
	(0.73)	(-0.88)	(1.39)
SOE	-0.189***	-3.923***	-1.058***
	(-10.76)	(-6.40)	(-2.72)
ACH	-0.069**	1.085	1.095***
	(-2.49)	(1.13)	(3.07)
BIG4	0.556***	-6.333***	-0.097
	(18.79)	(-6.15)	(-0.15)
LOPINION	0.098**	4.692***	3.708***
	(1.98)	(2.73)	(11.53)
_cons	6.273***	101.259***	
	(40.51)	(18.79)	
/.			
cut1			5.355*
			(1.80)
cut2			6.487**
			(2.18)
cut3			8.912***
			(2.97)
N	3469	3469	3469
YEAR	Yes	Yes	Yes
IND	Yes	Yes	Yes
R^2^	0.996	0.060	
ar2			

*** p<0.01

** p<0.05

* p<0.1

The regression outcomes, upon substituting the explanatory variables, align with the primary regression findings, reinforcing the inference that abnormal resignations of independent directors may lead to elevated audit fees. However, the likelihood of significant audit delays or auditors rendering outright non-standard audit opinions appears to be less probable.

### (v) Further studies

To investigate the impact of abnormal departures of independent directors on auditors’ decision-making, this study gathered a dataset of 3,752 publicly issued audit reports as of April 30, 2023. Among these reports, 231 were accompanied by non-standard audit opinions, including 106 cases with an unqualified opinion plus a matter paragraph, 90 cases with a qualified opinion or a qualified opinion plus a matter paragraph, and 35 cases where the auditors were unable to express an opinion. By examining the abnormal resignation of sole directors in listed companies that received non-standard opinions in 2022 and the abnormal departure of sole directors from companies that obtained non-standard opinions, the following findings were identified:

First, examining the prevalence of abnormal resignations of independent directors in 2021 or 2022, the percentage of listed companies that received non-standard audit opinions in the respective years of an independent director’s departure was 14.55% and 14.53%, which are nearly identical. However, for listed companies experiencing abnormal resignations of independent directors in both 2021 and 2022, the percentage of receiving non-standard audit opinions is 23.21%, significantly higher than that of the single year of departure. This suggests that following the Kangmei incident, when a listed company initially experiences the departure of an independent director, auditors may not perceive it as a material misstatement risk. However, should there be successive abnormal resignations of independent directors, auditors may become more vigilant, seeking additional information about the company, thus increasing the likelihood of issuing a non-standard opinion (Table 8).

**Table 8:** Percentage of non-standard opinions of companies with departing sole directors.

vintages	2021	2022	Separated in years 21 and 22
Number of companies with departures of sole directors	536	358	56
Number of non-standard comments received	78	52	13
percentage	14.55%	14.53%	23.21%

Secondly, comparing the percentages of companies receiving non-standard opinions in 2021, 2022, and in both years without abnormal resignations of independent directors (Table 9), it becomes evident that the likelihood of a listed company experiencing an abnormal resignation of an independent director receiving a non-standard opinion is significantly higher than that of companies without such departures, particularly in consecutive years with such departures, where the likelihood of receiving a non-standard opinion increases. Comparative analysis indicates that following the Kangmei incident, abnormal resignations of independent directors in listed companies send certain signals to auditors, prompting them to exercise increased diligence and thereby raising the likelihood of issuing non-standard opinions to those companies. This likelihood is even greater in cases of continuous resignations over multiple years.

**Table 9:** Percentage of non-standard opinions for companies without a sole director leaving the company.

vintages	2021	2022	Separated in years 21 and 22
Number of companies with no sole director departures	4236	3394	3081
Number of non-standard comments received	178	179	142
percentage	4.20%	5.27%	4.61%

Thirdly, a comparative analysis between the proportions of listed companies with abnormal independent director departures receiving non-standard opinions versus standard unqualified opinions demonstrates that the former is significantly higher for companies with such departures in either a single year or over consecutive years, with the proportion being even higher for consecutive years of abnormal departures (Tables 10 and 11).

**Table 10:** Percentage of sole director departures at companies that received a non-standard audit opinion.

vintages	2021	2022	Non-standard in 21 and 22 years
Number of companies receiving non-standard comments	256	231	141
Number of departures of sole directors	78	52	68
proportions	30.47%	22.51%	48.23%

**Table 11:** Percentage of departures of sole directors of companies receiving a standard unqualified audit opinion.

vintages	2021	2022	21, 22 average annual standards
Number of companies receiving standard comments	4516	3521	3162
Number of departures of sole directors	458	306	555
proportions	10.14%	8.69%	17.55%

Fourth, a comparison of the financial indicators of 231 listed companies that received non-standard audit opinions in 2021 and 2022 reveals that the 2022 financial indicators are generally worse than those of 2021 across three dimensions: the companies’ operating capabilities, solvency, and profitability (Table 12).

**Table 12:** Comparative analysis of financial indicators of companies resigned by sole directors in 22 years.

Financial Indicators	managerial ability		solvency			profitability	
	Current asset turnover ratio	Total asset turnover	flow ratios	Cash flow ratios	gearing	Net assets earnings yield (finance)	Business net profit margin
2021	0.96	0.47	1.66	-0.01	0.60	-0.80	-0.50
2022	0.95	0.43	1.58	-0.03	0.68	-0.49	-1.11

In summary, both from the standpoint of listed companies receiving non-standard opinions associated with the abnormal departure of independent directors and from the viewpoint of those associated with the abnormal departure of sole directors, the statistical analysis results indicate that the abnormal departure of independent directors exerts an influence on auditor decision-making, leading to deviations from the aforementioned regression results. This could be attributed to the regression analysis being supported by only a single year of data. It remains crucial to monitor the trend of abnormal departures of independent directors continually.

## Discussion and conclusion

### (i) Discussion

This study, set against the backdrop of the Kangmei Pharmaceuticals case, explores the impact of abnormal resignations of independent directors on auditors’ professional judgment, analyzing three dimensions: audit pricing, audit input, and audit opinions. The findings indicate that abnormal resignations of independent directors lead auditors to increase their risk assessment of clients, thereby influencing audit pricing. However, such resignations do not significantly impact audit input or audit opinions. These findings align with those of Cao et al. (2022), who also observed that resignations of independent directors heighten auditors’ risk alertness, prompting adjustments in audit strategies [35]. This paper distinguishes itself from existing research by specifically investigating the link between independent directors’ resignations and auditors’ professional judgment, supported by quantitative analysis results. While Salau et al. (2017) examined the relationship between independent directors’ resignations and corporate governance flaws, they did not delve into how these resignations specifically affect auditors’ judgments [36]. Notably, Huang (2023) discussed how media reports influence auditors’ professional judgment [37]. Although this paper does not directly focus on media influence, resignations of independent directors often come with extensive media coverage, which may indirectly affect auditors’ risk perception and professional judgment.

The results of this study suggest that while resignations of independent directors indeed heighten auditors’ alertness to potential risks, auditors may mitigate these risks through other mechanisms, such as enhanced risk assessment during the audit process, rather than necessarily increasing audit input or altering audit opinions. Moreover, by providing empirical analysis, this paper enriches the literature on the role of independent directors and the relationship with auditors’ professional judgment, offering new perspectives for both audit practice and theoretical research. Starting from the specific angle of independent directors’ resignations, this study deepens understanding of the factors influencing auditors’ professional judgment, revealing the intrinsic link between the resignations of independent directors and auditors’ professional judgment. It provides new evidence for theoretical research in corporate governance and auditing. The findings offer empirical support for auditors’ professional judgments in the face of independent directors’ resignations, helping auditors better identify and respond to potential risks triggered by such resignations in their actual audit work. A systematic analysis of the impact of independent directors’ resignations can inform the formulation of relevant regulatory policies and optimization of corporate governance structures, especially in strengthening the role of independent directors and improving audit quality.

### (ii) Conclusion

This study, grounded in the context of the Kangmei Pharmaceuticals case, investigates whether the abnormal resignations of independent directors influence auditors’ professional judgment. Through a quantitative analysis of the relationship between the abnormal resignation of independent directors and audit pricing, audit input, and audit opinions, the research reveals the mechanisms through which independent directors’ resignations impact auditors’ professional judgment. Controlling for other variables that might affect the outcomes, the study tested three hypotheses with the following results:

There is a positive correlation between audit fees and the resignation of independent directors, with a coefficient of 6.486, validating Hypothesis H1. This suggests that audit fees increase in companies experiencing abnormal resignations of independent directors. The abnormal resignation of independent directors raises auditors’ concerns about the risk of misstatements in the client’s financial reports, indicating that auditors adjust audit pricing to reflect the potential risks caused by independent directors’ resignations. However, the resignations do not significantly impact audit input and audit opinions, possibly because auditors mitigate this risk through other mechanisms, such as strengthening risk assessments and the execution of audit procedures.

There is no significant relationship between audit delay, audit opinions, and the resignation of independent directors, failing to validate Hypotheses H2 and H3. Hypothesis 2 explored whether the abnormal resignation of independent directors leads to increased audit input by auditors, potentially causing audit delays. According to the empirical analysis, the impact of abnormal resignations of independent directors on audit input is not significant. This means that although the resignation of independent directors might alert auditors to potential risks, it is not sufficient to cause a significant increase in audit input or result in audit delays. This could be because auditors may manage these risks through other audit strategies and procedures rather than simply increasing time investment. Hypothesis 3 examined whether the abnormal resignation of independent directors would make auditors more inclined to issue non-standard audit opinions. The relationship between the abnormal resignation of independent directors and the issuance of non-standard audit opinions by auditors is not significant. This suggests that while the abnormal resignation of independent directors may be seen by auditors as a signal of potential issues in financial reporting, it does not directly lead to more frequent issuance of non-standard audit opinions. This may reflect auditors taking a more comprehensive and cautious approach in assessing the risks of enterprises in the context of independent directors’ resignations.

This study enriches the existing literature on the relationship between the resignation of independent directors and audit quality, revealing the specific pathways through which independent directors’ resignations impact auditors’ professional judgment. This provides a new perspective on how auditors respond to changes in corporate governance, with significant theoretical and practical implications for enhancing corporate governance practices and audit quality. Considering the complex relationship between the abnormal resignation of independent directors and auditors’ professional judgment, future research is encouraged to explore this relationship’s dynamics and underlying mechanisms from a broader perspective, including corporate governance structure, market environment, legal systems, etc. Additionally, research should focus on the long-term impact of independent directors’ resignations on audit behavior and how to optimize audit practices and improve corporate governance quality.

## Supporting information

S1 Dataset (XLSX)
